# Comparison of neutralization potency across passive immunotherapy approaches as potential treatments for emerging infectious diseases

**DOI:** 10.1016/j.heliyon.2023.e23478

**Published:** 2023-12-16

**Authors:** Hossein Ranjbaran, Yahya Ehteshaminia, Mohammadreza Nadernezhad, Seyedeh Farzaneh Jalali, Farhad Jadidi-Niaragh, Abdol Sattar Pagheh, Seyed Ehsan Enderami, Saeid Abedian Kenari, Hadi Hassannia

**Affiliations:** a Immunogenetics Research Center, School of Medicine, Mazandaran University of Medical Sciences, Sari, Iran; b Department of Immunology, School of Public Health, Tehran University of Medical Sciences, Tehran, Iran; c Division of Microbiology, Department of Pathobiology, School of Public Health, Tehran University of Medical Sciences, Tehran, Iran; d Department of Hematology, Faculty of Allied Medicine, Kerman University of Medical Sciences, Kerman, Iran; e Immunology Research Center, Tabriz University of Medical Sciences, Tabriz, Iran; f Infectious Diseases Research Center, Birjand University of Medical Science, Birjand, Iran; g Department of Paramedicine, Amol School of Paramedical Sciences, Mazandaran University of Medical Sciences, Sari, Iran

**Keywords:** Emerging diseases, Passive immunotherapy, Plasma therapy, Neutralizing antibodies, Purified antibody

## Abstract

The use of passive immunotherapy, either as plasma or purified antibodies, has been recommended to treat the emerging infectious diseases (EIDs) in the absence of alternative therapeutic options. Here, we compare the neutralization potency of various passive immunotherapy approaches designed to provide the immediate neutralizing antibodies as potential EID treatments. To prepare human plasma and purified IgG, we screened and classified individuals into healthy, convalescent, and vaccinated groups against SARS-CoV-2 using qRT-PCR, anti-nucleocapsid, and anti-spike tests. Moreover, we prepared purified IgG from non-immunized and hyperimmunized rabbits against SARS-CoV-2 spike protein. Human and rabbit samples were used to evaluate the neutralization potency by sVNT. All vaccinated and convalescent human plasma and purified IgG groups, as well as purified IgG from hyperimmunized rabbits, had significantly greater levels of spike-specific antibodies than the control groups. Furthermore, when compared to the other groups, the purified IgG from hyperimmunized rabbits exhibited superior levels of neutralizing antibodies, with an IC50 value of 2.08 μg/ml. Additionally, our results indicated a statistically significant positive correlation between the neutralization IC50 value and the positive endpoint concentration of spike-specific antibodies. In conclusion, our study revealed that purified IgG from hyperimmunized animals has greater neutralization potency than other passive immunotherapy methods and may be the most suitable treatment of critically ill patients in EIDs.

## Graphical abstract

**Figure:**
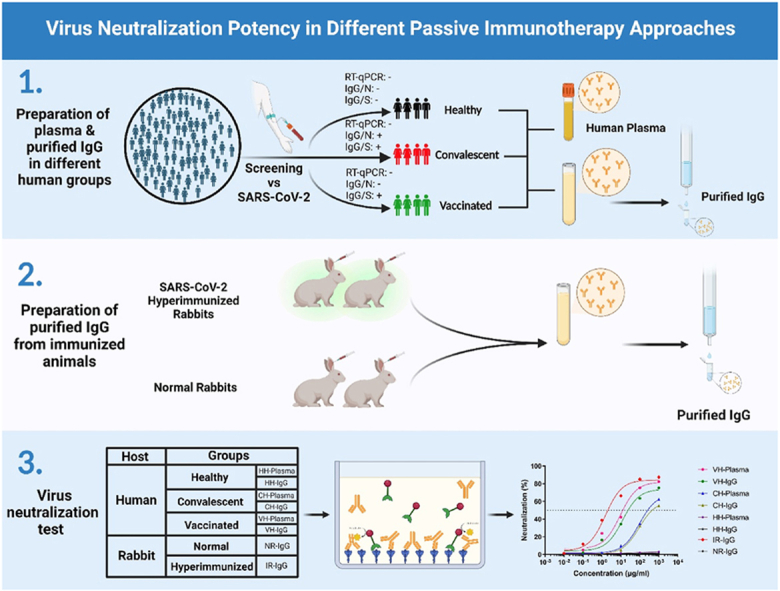

## 1 Introduction

Infectious diseases were responsible for billions of deaths before 1980 [1]. Many infectious diseases have been brought under control, and their associated mortality rates have reduced significantly thanks to developments in medical treatment, public health interventions, and countermeasures (diagnostics, therapeutics, and vaccines) [2]. However, emerging infectious diseases (EIDs), caused by unknown pathogens with high case-fatality ratios and contagiousness that carry the risk of becoming pandemic, is a primary concern for global health systems. Moreover, there is a limited scope for preventing, treating or controlling EIDs [3,4]. The 21st century has witnessed the emergence of several landmark events in the field of EIDs including the SARS (2003), Marburg (2004), H1N1 influenza (2009), MERS (2012), Ebola (2013), Zika (2016), Lassa fever (2018) and COVID-19 (2019) which have caused widespread morbidity and mortality, health care disruptions, economic and societal costs, and human suffering [5,6].

Many studies have demonstrated that the most effective approach to combat EIDs is the use of immunotherapy, which can be categorized to active and passive immunotherapy. Active immunotherapy involves artificial immunization with specific antigens by vaccination to stimulate the immune response, whereas passive immunotherapy confers immunity through the adoptive transfer of pathogen-specific antibodies [7,8]. While vaccination can provide durable and protective immunity against EIDs, human vaccine approval is a lengthy process [9]. For instance, the first vaccine was approved for emergency use in the midst of the COVID-19 pandemic in December 2020, whereas the pandemic had begun about a year prior; during this time, the pandemic had tragically resulted in the loss of 8.1 million lives, according to WHO statistics [10,11].

On the other hand, since begun of pandemic incidence until first vaccine approval when other therapeutic options are unavailable, passive immunotherapy is the best method for treating critically ill patients because it can induce immediate immunity in individuals [12]. Passive immunotherapy utilizes polyclonal and monoclonal antibodies. The monoclonal antibodies are produced in laboratories, while polyclonal antibodies are derived from immunized humans and animals. Despite the widespread use of monoclonal antibodies in a variety of infectious and non-infectious diseases, the process of producing and approving these antibodies is time-consuming, making them inappropriate for use in EIDs when the pathogen is unknown or when the pathogen's neutralizing epitopes have not been identified. In contrast, the use of polyclonal antibodies is preferred in new EIDs due to their low cost, large scale, rapid production, and recognition of a broad spectrum of epitopes, which reduces the pathogen's chances of escaping mutation [[13], [14], [15]].

Human-derived polyclonal antibodies have been used in post-exposure prophylaxis of rabies, hepatitis B, and tetanus; removal of the foreign agent prior to activation of the individual's immune system (as in using anti-Rh antibodies in Rh-negative women who have given birth to Rh-positive infants); and immunodeficient patients (such as those with Bruton agammaglobulinemia) [[16], [17], [18]]. Animal-derived polyclonal antibodies have been utilized to neutralize snake and scorpion venoms, as well as diphtheria and botulinum toxins, and to prevent acute rejection after organ transplantation through immunosuppressive treatment using anti-thymocyte globulin [[19], [20], [21]].

Plasma or purified IgG from convalescent individuals or purified IgG derived from animals can be used to prepare polyclonal antibodies for passive immunotherapy. Also, when the vaccine becomes available to the public, the use of plasma and purified IgG from vaccinated individuals can be an alternative option to treat critically ill patients and immunocompromised individuals. Despite the widespread use of various passive immunotherapy approaches, the optimal treatment for EIDs has yet to be determined. In this study, we will investigate the neutralization potency of plasma and purified IgG from convalescent and vaccinated individuals, as well as purified IgG from rabbits, against SARS-CoV-2.

## 2 Material and methods

### 2.1 Collection of human sera and plasma

In a cross-sectional study conducted between March 2020 and July 2021, 100 volunteers without underlying diseases provided serum samples with negative SARS-CoV-2 RT-PCR results. The volunteers were screened and classified into three groups of healthy, convalescent, and vaccinated, using the enzyme-linked immunosorbent assay (ELISA) for anti-nucleocapsid (anti-N) (Pishtazteb, Tehran, Iran) and anti-spike (anti-S) (Sinabiotech, Tehran, Iran) antibodies. Human samples collected prior to the outbreak of COVID-19 were also used as negative controls (healthy group). Four individuals were selected from each group based on high immunogenicity criteria (two doses of the ChAdOx1 nCoV-19 vaccine in the vaccinated group) and age and gender matching (in all three groups). Subsequently, 5 mL of citrate anticoagulant-containing blood and 5 mL of non-anticoagulant-containing blood were collected from these individuals. Serum and plasma samples were pooled separately and stored at −80 °C until use. Prior to enrolling participants in the study, it was ensured that each individual provided their consent by signing an informed consent form. Each participant was thoroughly informed about the aims of the study, the potential advantages and risks that might be involved. The Ethics Committee of Mazandaran University of Medical Sciences, Iran, approved the research project (IR.MAZUMS.REC.1399.106).

### 2.2 Purification of human polyclonal IgG and assessment of the reactivity of collected plasma and purified IgG with SARS-CoV-2 spike protein

In order to purify human polyclonal IgG, selected individuals' sera were diluted 1/20 in PBS and filtered through a 0.45-μm filter. The diluted sera were then injected into a Hi-Trap protein A column (GE Healthcare Life Sciences, Uppsala, Sweden). The concentration of purified IgG was measured using the extinction coefficient following dialysis in PBS. Before the reactivities of plasma and purified IgG were compared with SARS-CoV-2 spike protein, the total IgG content of plasma samples was determined. All samples, including plasma and purified IgG, were normalized by diluting them to 1 mg/ml of total IgG in PBS. After normalization of human plasma and purified IgG in different groups, endpoint titration ELISA was used to evaluate the antibody's reactivity against SARS-CoV-2 spike protein. Briefly, 5 μg/ml of spike protein was used to coat 96-well plates (SPL, Life Science, Korea) and kept at 4 °C overnight. Unbound antigens were removed by washing three times with PBST (phosphate-buffered saline containing Tween detergent). Then, coated plates were blocked with 250 μL of blocking buffer (5 % BSA in PBST) at room temperature for an hour, washed 3 times with washing buffer. The samples were subsequently titrated against the coated antigen. An HRP-conjugated goat anti-human IgG (Abcam, ab6858) was added and incubated at 37 °C for 60 min. Plates were subsequently washed, and tetramethylbenzidine (TMB) substrate (Pishtazteb, Tehran, Iran) was added. The reaction was terminated by adding 1 M H2SO4, and the optical density (OD) was measured at 450/630 nm using an ELISA reader (BioTek, Winooski, VT, USA). The positive endpoint titration for human plasma and purified IgG samples was determined by samples with an OD value above the cutoff point (mean plus twofold standard deviation of healthy human plasma and purified IgG as a negative control).

### 2.3 Preparation of purified IgG from hyperimmunized rabbits and assessment of the reactivity of purified IgG with SARS-CoV-2 spike protein

As described in our previous study [22], we intramuscularly immunized two female New Zealand white rabbits (Pasteur Institute of Tehran, Iran) with 20 μg recombinant S1-FC fusion protein accompanied by 10 μg of CpG adjuvant (Bioneer, Republic of Korea) four times with two administration per week. Two weeks after the fourth immunization rabbit sera and also sera of two non-immunized rabbits was collected. Immunized and non-immunized rabbit IgG (IR-IgG and NR-IgG, respectively) were purified using a protein A chromatography column following the dilution of sera in PBS. After the concentration of purified IgG was measured, the antibody's reactivity was calculated by endpoint ELISA titration according to the previous ELISA section, except that HRP-conjugated mouse anti-rabbit IgG (Abcam, ab99697) was used for secondary antibody detection.

### 2.4 Surrogate virus neutralization test (sVNT)

The SARS-CoV-2 sVNT kit (Pishtazteb, Tehran, Iran) was used in accordance with the manufacturer's instructions to determine the neutralizing antibodies potency of human and rabbit samples. Briefly, normalized samples were serially diluted with sample dilution buffers from 1000 to 0.01 μg/ml of total IgG and subsequently mixed with an equal volume of HRP-conjugated ACE2. Afterward, the solution was added to the pre-coated RBD wells and incubated at 37 °C for 30 min. Unbound HRP-conjugated ACE2 was washed away after incubation. Upon the addition of TMB, the OD value was measured at 450/630 nm using an ELISA reader. The percentage of inhibition was calculated using the following formula: (1 − sample OD value/average negative control OD value) × 100. In addition, the IC50 value was determined to represent the neutralizing antibodies potency.

### 2.5 Graphing and statistical analysis

GraphPad Prism (Version 9, San Diego, California, USA) was utilized for raw data analysis and graphing. The correlation between the neutralization IC50 value and positive endpoint concentration of spike-specific antibodies was analyzed by Spearman correlation coefficients. There were at least three repetitions of each experiment. Data were presented as means ± standard deviations. Differences were deemed statistically significant with *P < 0.05 and **P < 0.01, or when results exceeded the cutoff point (mean plus twofold standard deviation of the negative control sample).

## 3 Results

### 3.1 Screening and categorization of healthy, convalescent, and vaccinated individuals

**Fig. 1:**
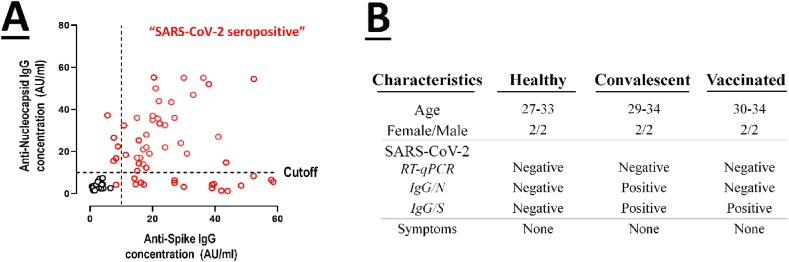
**A)** Screening and classification of individuals into three groups using anti-S and N protein antibodies by ELISA (Red circles indicate the level of antibodies in unvaccinated and vaccinated individuals after the COVID-19 pandemic; black circles represent the level of antibodies in healthy individuals prior to the COVID-19 pandemic) **B)** Demographic characteristics of convalescent, vaccinated, and healthy individuals selected for subsequent analysis. (For interpretation of the references to colour in this figure legend, the reader is referred to the Web version of this article.)

One hundred volunteers with negative results for SARS-CoV-2 RT-PCR were tested with an ELISA to determine their antibody levels against the S and N proteins. Positive samples for anti-S and anti-N were deemed convalescent, whereas positive samples for anti-S but negative for anti-N were deemed vaccinated. In addition, samples with positive results for anti-S and anti-N that were vaccinated against SARS-CoV-2 according to a questionnaire were excluded from the study. Likewise excluded were samples with negative results for anti-S and anti-N that were collected after the COVID-19 pandemic. Human samples collected before the COVID-19 pandemic were also used as a negative control (healthy group) (Fig. 1A). On the basis of the questionnaire and ELISA results, four individuals from each group were selected for further analysis; their characteristics are depicted in Fig. 1B.

### 3.2 Evaluation of endpoint titration of spike-specific polyclonal IgG from human and rabbits samples

**Fig. 2:**
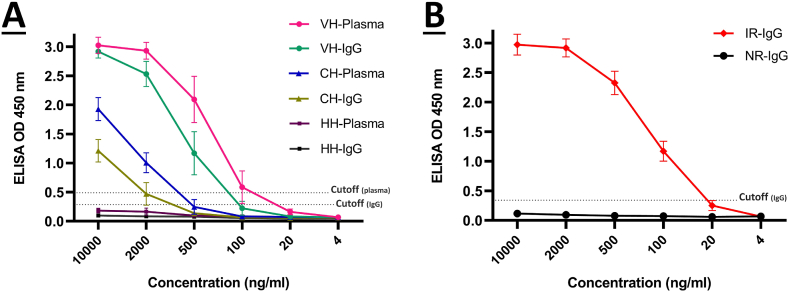
Endpoint titration of spike-specific IgG from human and rabbit samples. **A)** Normalized samples of human plasma and purified IgG were titrated 10,000–4 ng/ml. **B)** Purified IgG from rabbits were also titrated 10,000–4 ng/ml. Samples with two-fold standard deviations above the mean of the negative controls (cutoff) were considered positive endpoint titration. VH-Plasma: Vaccinated human plasma; VH-IgG: Vaccinated human purified IgG; CH-Plasma: Convalescent human plasma; CH-IgG: Convalescent human purified IgG; HH-Plasma: Healthy human plasma; HH-IgG: Healthy human purified IgG; IR-IgG: Immunized rabbit purified IgG; NR-IgG: Non-immunized rabbit purified IgG.

For each group's spike-specific endpoint titration, an indirect ELISA was conducted with sample dilutions ranging from 10,000–4 ng/ml. As illustrated in Fig. 2A, all vaccinated human plasma (VH-plasma), vaccinated human purified IgG (VH-IgG), and convalescent human plasma (CH-plasma) groups had OD values greater than the cutoff point. The cutoff point was determined using human control groups, healthy human plasma (HH-plasma), and healthy human purified IgG (HH-IgG). Positive endpoint titrations for VH-plasma, VH-IgG, CH-plasma, and CH-IgG were 81.7, 130, 979, and 1164 ng/ml, respectively. The results indicate that vaccinated individuals have higher levels of spike-specific antibodies in their plasma and purified IgG than convalescent individuals. In addition, endpoint titration was performed on purified rabbit antibodies. As depicted in Fig. 2B, the OD values of IR-IgG were greater than the cutoff (with 27.3 ng/ml positive endpoint titration).

### 3.3 Comparison of viral neutralization potency in human and rabbit samples

**Fig. 3:**
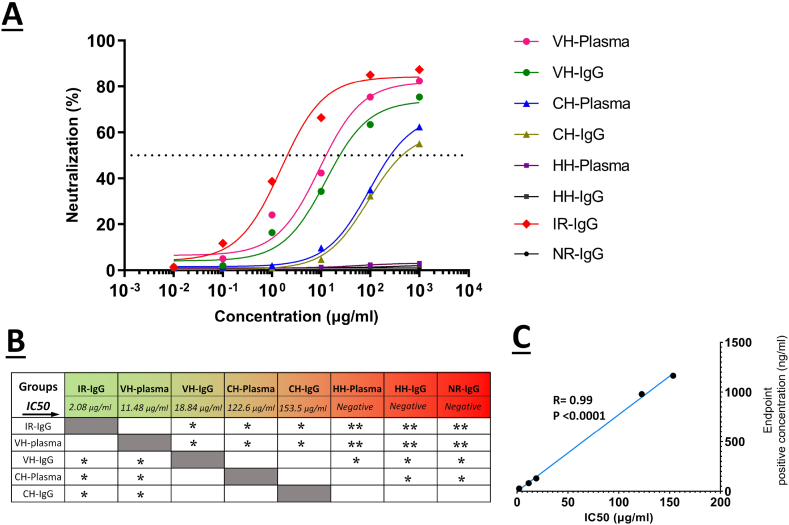
Comparison of neutralization potency across passive immunotherapy approaches. **A)** The neutralizing potency of each group was demonstrated by the sVNT curve. **B)** The IC50 values for neutralization were determined using the sVNT curve, and statistical comparisons were conducted for all groups. **C)** Two-tailed Spearman correlation test was conducted between positive endpoint concentration of spike-specific antibodies and IC50 values in each group. *P < 0.05 and **P < 0.01 were considered as statistically significant differences. VH-Plasma: Vaccinated human plasma; VH-IgG: Vaccinated human purified IgG; CH-Plasma: Convalescent human plasma; CH-IgG: Convalescent human purified IgG; HH-Plasma: Healthy human plasma; HH-IgG: Healthy human purified IgG; IR-IgG: Immunized rabbit purified IgG; NR-IgG: Non-immunized rabbit purified IgG; IC50: 50 % inhibitory concentration.

The sVNT test was performed to compare the levels of neutralizing antibodies in human and animal samples. The response of neutralizing antibodies was greater in hyperimmunized rabbits and vaccinated humans than in all other groups (Fig. 3A). In addition, the neutralization IC50 values for the VH-plasma, VH-IgG, CH-plasma, CH-IgG, and IR-IgG groups were 11.48, 18.84, 122.6, 153.5, and 2.08 μg/ml, respectively. As expected, no neutralizing antibody response was observed in the control groups (Fig. 3B). Moreover, a two-tailed Spearman correlation test was conducted between the positive endpoint concentration of spike-specific antibodies and the IC50 values of each group, which reveals a direct and significant correlation between the spike-specific antibody level and that of neutralizing antibody in each group (r = 0.99; p < 0.001; Fig. 3C).

## 4 Discussion

Multiple methods can be used to prepare passive immunotherapy from human and animal sources. The utilization of convalescent or vaccinated human plasma is a common passive immunotherapy method [14]. According to a study by Hung et al., the administration of convalescent plasma to influenza A (H1N1) patients resulted in a 34 % decrease in mortality compared to the conventional treatment group [23]. Using purified antibodies derived from humans is another method of passive immunotherapy. Huygens et al. demonstrated that human-derived purified antibodies reduce by 68 % the risk of severe COVID-19 infection in immunocompromised patients compared to the control group [24].

Nonetheless, these two approaches require collecting large quantities of pathogen-specific antibodies from human volunteers, whose neutralizing antibody titers may vary. While another passive immunotherapy method employing animal-derived purified antibodies can rapidly produce pathogen-specific antibodies on a large scale [14]. Today, there is a tendency to use animal-derived purified antibodies because they can be produced on a large scale without the need for human volunteers [14].

Despite the widespread interest in passive immunotherapy, no study has yet compared the neutralization potency of different passive immunotherapy methods. In this study, we collected plasma and IgG from healthy, convalescent, and vaccinated individuals (Fig. 1), as well as IgG from non-immunized and hyperimmunized rabbits against SARS-CoV-2 spike protein. We used an endpoint ELISA titration to evaluate the spike-specific antibody levels. The results showed that in comparison to the control groups, both artificial (VH-plasma, VH-IgG, and IR-IgG) and natural (CH-plasma and CH-IgG) immunization approaches induced a significant spike-specific antibody response (Fig. 2A and B). These antibodies were significantly higher in hyperimmunized animals and vaccinated individuals than in convalescent individuals, attributable to the injection of booster doses and potent adjuvants. Moreover, despite identical total IgG concentrations in all samples (as described in section 2.2, plasma and purified IgG were normalized to 1 mg/ml of total IgG), the results demonstrated that plasma has a slightly higher reactivity to spike protein than purified IgG. This difference may be attributable to antibody destruction resulting from structural changes, aggregation, and pH variation during the purification procedure [[25], [26], [27]].

The sVNT test was conducted to determine the level of neutralizing antibodies among different groups. The results demonstrated that the neutralizing antibody response of hyperimmunized rabbits is significantly greater than other groups (Fig. 3A and B). This distinction may stem from the presence of a plateau level of neutralizing antibodies in hyperimmunized rabbits resulting from a higher number of immunizations and potent adjuvants, compared to the variable levels of neutralizing antibodies in the vaccinated human group, which have not yet reached a plateau [22,28].

In addition, a significant positive correlation was observed between the neutralization IC50 value and the positive endpoint concentration of spike-specific antibodies, suggesting that an increase in the level of spike-specific antibodies corresponds to increased neutralizing antibodies (Fig. 3C). In line with our findings, Grunau et al. demonstrated a linear correlation between viral neutralizing antibody titers and anti-spike antibody levels in COVID-19-vaccinated individuals [29].

**Fig. 4:**
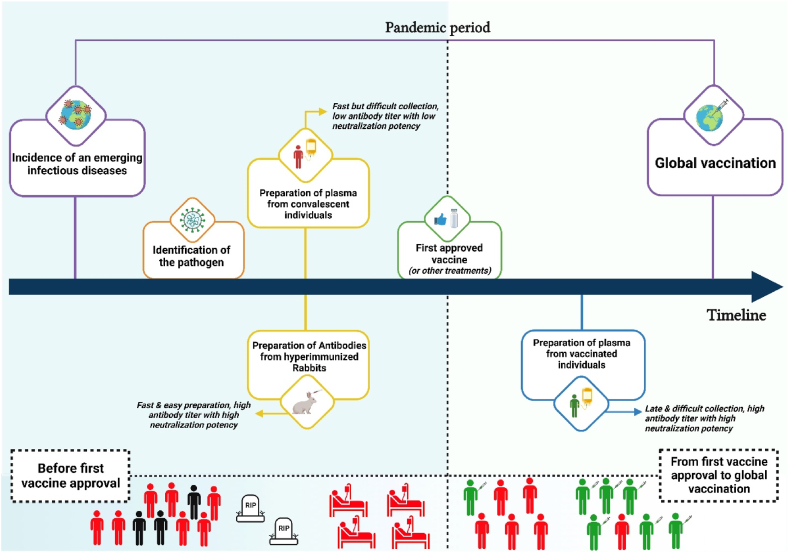
Schematic depiction of the use of immunotherapy from the onset of the EID to the conclusion of global vaccination. As depicted in the graph, between the onset of the pandemic and the release of the first vaccine, EID can cause a large number of deaths, and the only treatment for critically ill patients is passive immunotherapy. According to the findings of this study, the use of purified IgG from hyperimmunized animals can provide the highest level of neutralizing antibodies in the shortest time after the onset of a pandemic.

As illustrated in Fig. 4, animal-derived purified antibodies can be employed to combat new EIDs. These antibodies can be produced rapidly on a large scale and contain the highest concentration of neutralizing antibodies. By increasing the neutralization potency of antibodies, the cost of treatment is reduced and open up alternative routes of administration possible that extend antibody effective half-life [30]. However, it is more challenging to prepare antibodies with high neutralization potency in vaccinated individuals due to the lengthy process of human vaccine approval. Therefore, animal-derived purified antibodies appear more appropriate than other passive immunotherapy methods for EIDs [14].

One further outcome of this research is the possibility of employing purified antibodies instead of plasma therapy. Multiple studies have demonstrated that plasma contains over a thousand proteins, of which only 20 % are antibodies and less than 2 % (∼200 μg/ml) of these antibodies are pathogen-specific [15,31,32]. The overabundance of proteins in plasma is associated with known risks, such as allergic reactions (ranging from mild to life-threatening anaphylaxis) and transfusion-related acute lung injury (TRALI), as well as a transfusion-associated circulatory overload (TACO). Purified IgG, on the other hand, has a purity of over 96 %, allowing for smaller injection volumes and easier storage and transport [[33], [34], [35]]. Notably, unlike plasma therapy, purified antibodies do not require blood group matching, and the risk of transmitting blood-borne diseases is minimized [35,36].

Currently, several animal-purified polyclonal antibodies are used therapeutically in patients for such applications as preventing acute organ rejection after transplantation (Atgam® and Thymoglobulin®), removing toxic levels of digoxin (Digifab®), neutralizing crotalid snake venoms (CroFab®), and treating symptomatic botulism (BAT®). These products have shown a promising safety and efficacy record, particularly in critical situations where the benefits of treatment far exceed the potential risks [[37], [38], [39], [40]]. To mitigate the risk of hypersensitivity reactions to xenogenic antibodies, one approach is to use enzyme digestion to generate Fab fragments instead of using whole IgGs. However, a more appropriate method is to utilize transchromosomic animals. This technology allows for the production of fully human antibodies against pathogens [14].

There are a few potential limitations to this study. First, affinity-purified specific antibodies were not used in this study, which is deemed essential for future research. Second, because our laboratory did not have access to biosafety level 3, we were unable to use the gold standard conventional virus neutralization test [41]. Our research indicates that the purified antibodies from hyperimmunized animals may be a promising strategy for treating critically ill patients in new EIDs than other passive immunotherapy methods. We hope that using a more appropriate passive immunotherapy method, we will be able to control EIDs during pandemic outbreaks and reducing their associated mortality.

## Funding

Mazandaran University of Medical Sciences provided the funds for this research project (Grant No. 7306).

## Data availability statement

Data will be made available on request. No data associated with this study has been deposited into a publicly available repository.

## Ethics declarations

This study was reviewed and approved by the Research Ethics Committee of Mazandaran University of Medical Sciences (approval ID: IR. MAZUMS.REC.1399.106). All participants provided informed consent to participate in the study.

## Additional information

No additional information is available for this paper.

## CRediT authorship contribution statement

**Hossein Ranjbaran:** Writing – review & editing, Methodology, Investigation. **Yahya Ehteshaminia:** Writing – original draft, Methodology, Investigation. **Mohammadreza Nadernezhad:** Writing – original draft, Methodology, Investigation. **Seyedeh Farzaneh Jalali:** Methodology, Investigation. **Farhad Jadidi-Niaragh:** Supervision, Formal analysis, Data curation, Conceptualization. **Abdol Sattar Pagheh:** Supervision, Formal analysis, Data curation, Conceptualization. **Seyed Ehsan Enderami:** Supervision, Formal analysis, Data curation, Conceptualization. **Saeid Abedian Kenari:** Supervision, Formal analysis, Data curation, Conceptualization. **Hadi Hassannia:** Writing – review & editing, Supervision, Project administration, Methodology, Investigation, Funding acquisition, Data curation, Conceptualization.

## Declaration of competing interest

The authors declare that they have no known competing financial interests or personal relationships that could have appeared to influence the work reported in this paper.
